# Fabrication of Unique Magnetic Bionanocomposite for Highly Efficient Removal of Hexavalent Chromium from Water

**DOI:** 10.1038/srep31090

**Published:** 2016-08-09

**Authors:** Yunlei Zhong, Xun Qiu, Dongyun Chen, Najun Li, Qingfeng Xu, Hua Li, Jinghui He, Jianmei Lu

**Affiliations:** 1 College of Chemistry, Chemical Engineering and Materials Science, Collaborative Innovation Center of Suzhou Nano Science and Technology, Soochow University, Suzhou, 215123, China

## Abstract

Biotreatment of hexavalent chromium has attracted widespread interest due to its cost effective and environmental friendliness. However, the difficult separation of biomass from aqueous solution and the slow hexavalent chromium bioreduction rate are bottlenecks for biotechnology application. In this approach, a core-shell structured functional polymer coated magnetic nanocomposite was prepared for enriching the hexavalent chromium. Then the nanocomposite was connected to the bacteria via amines on bacterial (*Bacillus subtilis* ATCC-6633) surface. Under optimal conditions, a series of experiments were launched to degrade hexavalent chromium from the aqueous solution using the as-prepared bionanocomposite. Results showed that *B. subtilis*@Fe_3_O_4_@mSiO_2_@MANHE (BFSM) can degrade hexavalent chromium from the water more effectively (a respectable degradation efficiency of about 94%) when compared with pristine *B. subtilis* and Fe_3_O_4_@mSiO_2_@MANHE (FSM). Moreover, the BFSM could be separated from the wastewater by magnetic separation technology conveniently due to the Fe_3_O_4_ core of FSM. These results indicate that the application of BFSM is a promising strategy for effective treating wastewater containing hexavalent chromium.

Under natural conditions, the atmosphere, soil and water contains trace amounts of chromium compound. Hexavalent chromium mainly exists in forms of Cr_2_O_7_^2−^ and CrO_4_^2−^ which is highly mobile^1^, water soluble and toxic to all living organisms^2^. Because of high solubility, hexavalent chromium goes into the living cells easily and produces reactive oxygen species (ROS), resulting in serious oxidative injuries to cell constituents^3^. The main effects of hexavalent chromium for humans are dermatitis and aggressive reaction in lungs and nasal septum^4^,^5^. The maximum total chromium concentration in water body is limited to 0.1 mg/L according to EPA drinking water standards^6^,^7^,^8^,^9^. However, the concentration of hexavalent chromium is over 1000 times in the ordinary wastewater^9^,^10^,^11^,^12^. Unfortunately, chromium is widely used in numerous industrial processes, including leather tanning, pigment production, electroplating and ore refining^13^,^14^,^15^,^16^. In this context, if the industrial containing chromium wastewater could not be effectively addressed, it may lead to the contamination of natural water sources, and ultimately threatening human health^17^,^18^,^19^,^20^,^21^,^22^.

Because of this, far-ranging conventional methodologies have been used for water purification, including filtration and coagulation/sedimentation/flocculation, liquid extraction, chemical oxidation, membrane processes, and so on^23^,^24^,^25^,^26^. However, these methodologies have been proved to be inefficient and uneconomic for the treatment of hexavalent chromium^27^,^28^. To overcome these disadvantages, a great deal of attentions have been concentrated on microbial remediation strategy for hexavalent chromium contamination through sorption, accumulation and reorganization^1^,^29^, which is considered as low-cost and eco-friendliness comparing with chemical methods^30^,^31^. Up to date, multifarious bacteria have the ability of reducing hexavalent chromium to less toxic Cr(III) under aerobic or anaerobic conditions^32^,^33^,^34^,^35^,^36^. For example, *Bacillus sp*., *Ochrobactrum sp*., *Enterobacter sp*., *Pseudomonas sp*., *Pannonibacter sp*., *Arthrobacter sp*., *Acinetobacter sp*. and *Exiguobacterium sp*. are such functional bacteria^37^,^38^. Nevertheless, biological treatment also has its own drawbacks, for example, it will take several days or even weeks to acheive the complete reduction of hexavalent chromium (especially at low concentration) under optimal conditions^1^,^39^. Moreover, the difficult separation of bacteria from the treated wastewater also limits its application^39^. Therefore, it is necessary to develop new biotechnologies that can effectively achieve hexavalent chromium reduction and rapidly separate bacteria from the wastewater after the treatment^30^. Recently, magnetic nanocomposite was exploited for heavy metal ions reduction due to its high magnetic separation efficiency^39^,^40^,^41^. Moreover, silica was reported as an ideal protection layer for Fe_3_O_4_ NPs not only due to its high chemical stability and great biocompatibility but also its highly reactive surface^42^,^43^,^44^. In addition to these, polymers including polyacrylamide, polyaniline (PANI) and polyethylenimine had been taken increasing notice for heavy metal removal because of their high removal efficiency^45^,^46^,^47^. For instance, PANI, as a kind of conductive polymer, had been paid intensive attention for potential environmental applications because of its large surface area and abundant active sites^48^. Nowadays, many kinds of polymers had been coated on the surface of the easily separated materials including fibers^49^,^50^, sawdust, Fe_3_O_4_ nanoparticles, which showed excellent pollutants removal performance.

In this paper, a core-shell structured magnetic nanocomposite for efficient enrichment of chromium ions from water was first fabricated. Fe_3_O_4_ was selected as the core of nanomaterials due to its good magnetic separation efficiency. Then the Fe_3_O_4_ was coated with mesoporous silica (mSiO_2_), which have been used as promising adsorbents for the water remediation and provided advantages such as large surface area, high surface reactivity, and regular mesoporous structures. In addition, the efficacy of mesoporous silica in the adsorption process could be highly improved via surface functionalization with particular groups for adsorption of specific substances. Based on this assumption, the surface of mesoporous silica was modified with amine groups, and 4, 4-azo-bis(4-Cyanopentanoicchloride) (ABCPA, Fig. 1), an active radical initiator was grafted via these amine groups^51^. Functional polymer consisting 4-vinyl pyridine and N-(methacryloyloxy)-succinimide could encapsulate Fe_3_O_4_@mSiO_2_ via polymerization. Then, the nanocomposite was attached on the surface of bacteria through the coupling reaction between the N-(methacryloyloxy)-succinimide group of the nanocomposite and the amine group of the bacteria cells. Finally, magnetic bacteria systems were established to achieve rapid and effective degradation of hexavalent chromium and magnetic separation of bacteria.

## Results and Discussion

### Cr(VI) reduction by planktonic cells

**Figure 2:**
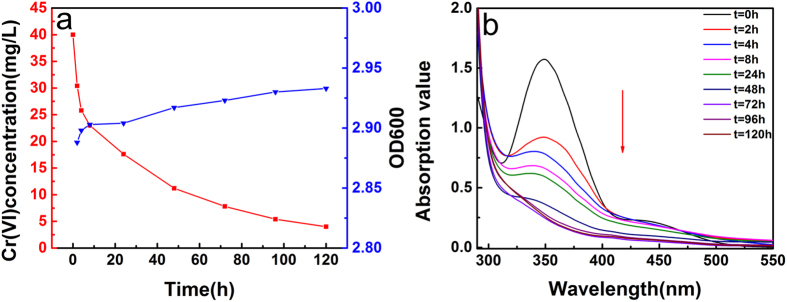
The reduction ability of Cr(VI) of the planktonic cells and OD600 of *B. subtilis* (**a**) (initial Cr(VI) concentration = 40 mg/L, volume of Cr(VI) solution = 50 mL, pH = 7 and T = 30 °C); UV-vis absorption spectrum of Cr(VI) degradation (**b**).

In the present study, we investigated the hexavalent chromium reduction by planktonic cells of *B. subtilis* in a glucose solution. Figure 2a shows that 40 mg/L of hexavalent chromium was decreased to approximately zero by planktonic cells of *B. subtilis* within 96 h, while the OD600 of *B. subtilis* can be seen an obvious increasement which corresponding with the quantity of the bacteria cells. The result showed that bacteria could subsist very well in the presence of hexavalent chromium. The UV-vis spectrum of Cr-diphenylcarbazide complex was shown in Fig. 2b. It could be clearly seen that the adsorption value changed a lot and even decrease to zero after 96 h. This result strongly demonstrates the biodegradation of hexavalent chromium, which further confirm the biodegrade ability of *B. subtilis*.

**Figure 3:**
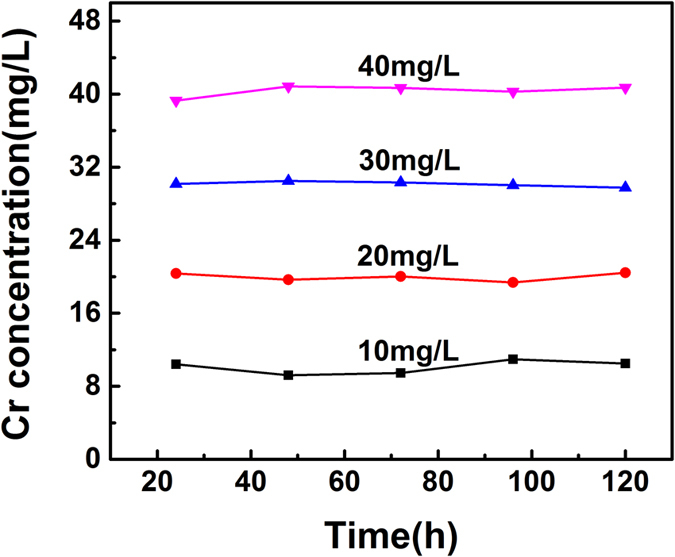
The total Cr concentration of 10 mg/L, 20 mg/L, 30 mg/L, 40 mg/L after degradation by *B. subtilis*.

To further investigate the degradation process of hexavalent chromium, the total chromium concentration of 10 mg/L, 20 mg/L, 30 mg/L and 40 mg/L after treated at different times were studied by atomic absorption spectrometry. As shown in Fig. 3, the total chromium concentration of 10 mg/L, 20 mg/L, 30 mg/L and 40 mg/L had no changes in the process of degradation even after 120 h. The above results indicated that the planktonic cells of *B. subtilis* only degrade hexavalent chromium without adsorption chromium ions.

**Figure 4:**
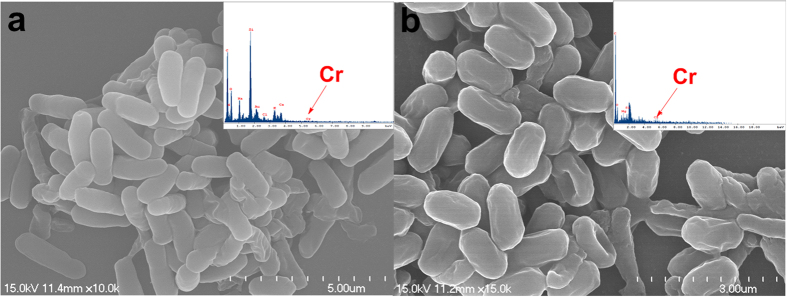
SEM images of *B. subtilis* and SEM-EDS of planktionic cells (**a**); SEM of Cr-loaded *B. subtilis* and SEM-EDS of Cr-load planktionic cells (**b**).

In order to illustrate the morphologic changes and detail distribution of degraded hexavalent chromium on planktonic cells, SEM was performed and the results were shown in Fig. 4. As can be seen in Fig. 4a, the long–rod shaped *B. subtilis* have a smooth surface, and the planktonic cells were plumb before the biodegradation of hexavalent chromium. After treated with 40 mg/L hexavalent chromium for 120 h, the surface of bacteria became rough and lean (Fig. 4b). EDS spectra demonstrated that a small amount of chromium was accumulated on the bacterial surfaces (Fig. 4b). This result further confirms that the planktonic cells of B. subtilis mainly degrade hexavalent chromium instead of adsorption.

### Characterization of nanoparticles

**Figure 5:**
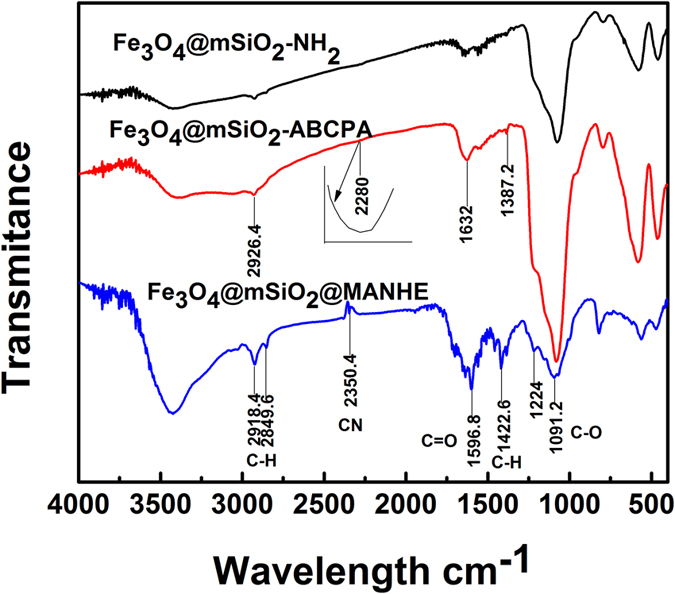
FT-IR spectra of Fe_3_O_4_@mSiO_2_-NH_2_, Fe_3_O_4_@mSiO_2_-ABCPA, FSM composites.

FT-IR spectra of Fe_3_O_4_@mSiO_2_-NH_2_, Fe_3_O_4_@mSiO_2_–ABCPA (4,4-Azobis(4-cyanovaleric acid)), FSM nanocomposites are displayed in Fig. 5. As shown in the spectrum of Fe_3_O_4_@mSiO_2_-NH_2_, the peak observed at 580 cm^−1^ was characteristic of the Fe-O vibration. The peaks at 1076 and 3432 cm^−1^ were from the stretching vibration of Si-O and Si-OH bonds, separately. After grafting of free-radical initiator on the Fe_3_O_4_@mSiO_2_ magnetic nanocomposites, two new peaks at 1387 cm^−1^ and 1632 cm^−1^ corresponding to C-H symmetric and asymmetric bending vibrations of methyl groups in ABCPA were observed. The observation of the characteristic absorption peak at 2280 cm^−1^ representing the C≡N stretching vibrations of ABCPA also demonstrates successfully grafting of free-radical initiator to Fe_3_O_4_@mSiO_2_ nanocomposites. On the spectrum of FSM, the absorption peaks at 2918 cm^−1^ and 1596 cm^−1^ are identified as C-H asymmetric stretching vibration and C=O stretching vibration of the grafted MANHE chains.

**Figure 6:**
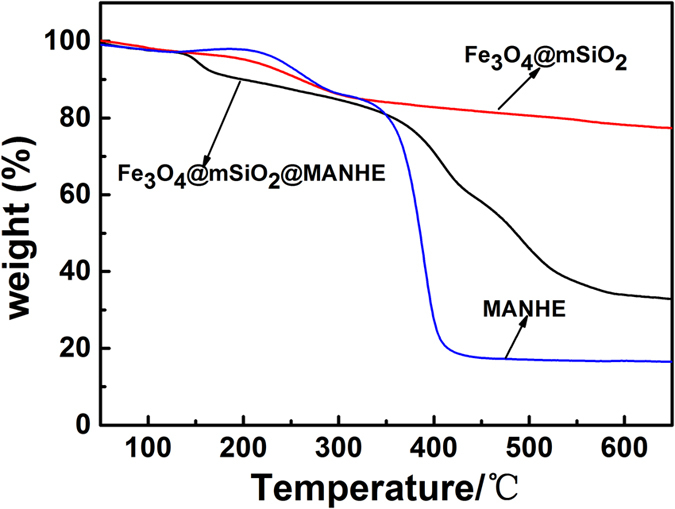
TGA curves of Fe_3_O_4_@mSiO_2_, FSM and MANHE.

Fe_3_O_4_@SiO_2_, FSM nanocomposites and MANHE were also characterized by TGA. As shown in Fig. 6, Fe_3_O_4_@SiO_2_ have about 20 wt.% weight loss in the range between 100 and 700 °C, which was corresponding to the loss of the functional groups such as OH groups on the surface of Fe_3_O_4_@SiO_2_. MANHE polymer showed about 82.5% weight loss of functional groups and carbon chain skeleton in the range between 100 and 700 °C. However, FSM nanocomposites showed about 68 wt.% weight loss after polymerization of 4-vinyl pyridine and N-(methacryloyloxy)-succinimide. All these results confirmed the successful polymer modification which was in good agreement with the FT-IR (Fig. 5).

**Figure 7:**
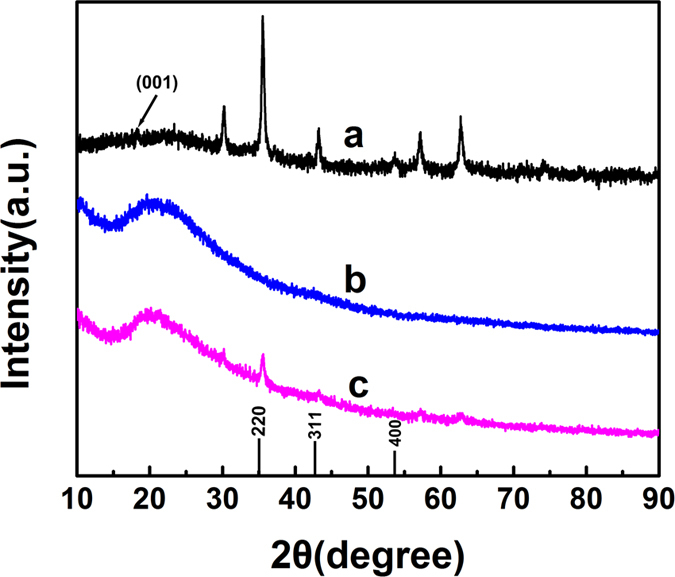
Wide-angle XRD patterns of FSM (**a**); *B. subtilis* (**b**); BFSM(**c**).

FSM, *B. subtilis* and BFSM were prepared under optimal conditions and then characterized by XRD. As can be seen in Fig. 7a, the characteristics of the crystal plane diffraction peaks (220, 311, 400) of Fe_3_O_4_ appeared at 35.2°, 41.7°, 50.6°, respectively. Meanwhile, the peak at 16 ∼ 36° indexed as (001) could be attribute to characteristic diffraction peaks of the amorphous SiO_2_. The results indicated that the crystal form of the nanoparticles did not change after the coating of MANHE. The XRD spectra of *B. subtilis* was shown in Fig. 7b, there are no obvious characteristic peaks. And the XRD spectra of BFSM were shown in Fig. 6c, an obvious peak at 35° was corresponding to the magnetic core. This result strongly indicated that the FSM have been successfully modified to the surface of bacteria. Moreover, the results of above XRD further support the results of FT-IR.

**Figure 8:**
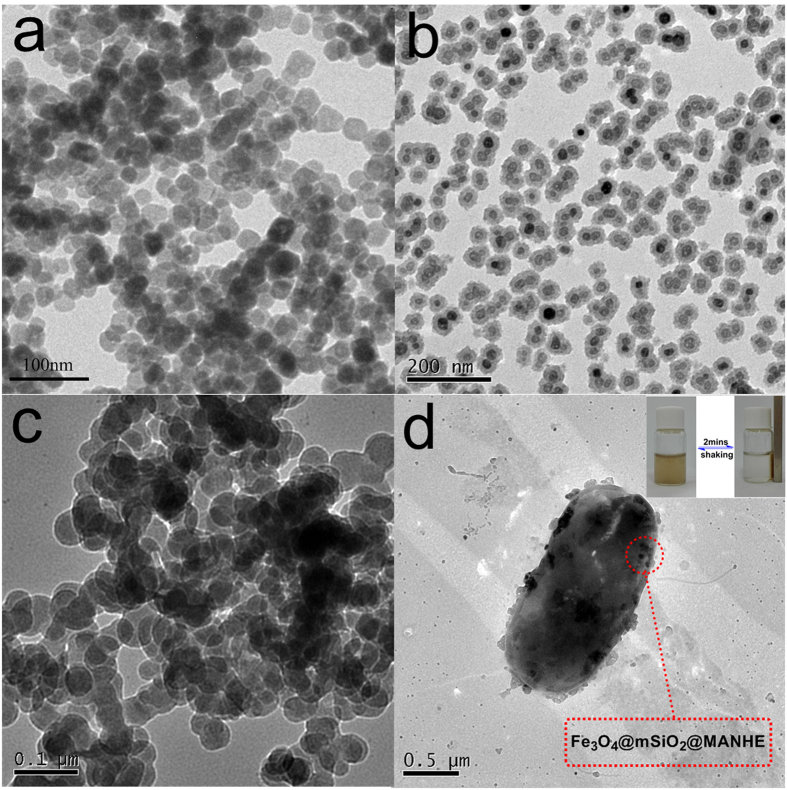
TEM images of Fe_3_O_4_ (**a**); Fe_3_O_4_@SiO_2_ (**b**); FSM (**c**); BFSM (**d**).

The structure and morphological features of magnetic nanoparticles were further examined by TEM (Fig. 8). The images of TEM obviously indicate that monodispersed microspheres with narrow size distribution were obtained for all the samples. As shown in Fig. 8a, the Fe_3_O_4_ synthesized by hydrothermal method had a uniform diameter at about 30 nm with excellent dispersion. In addition, from the images of Fe_3_O_4_@mSiO_2_ and FSM nanoparticles (Fig. 8b,c), the core-shell structure could be clearly distinguished owing to the different electron penetrability between Fe_3_O_4_, SiO_2_ and MANHE. As shown in Fig. 8b, Fe_3_O_4_@mSiO_2_ was spherical, and the thickness of the mesoporous silica layer was about 20 nm. As shown in the TEM image of FSM (Fig. 8c), the magnetic nanocomposites modified by polymer with large amounts of nitrogen remained spherial. The thickness of MANHE shell showed gray color was about 30 nm (Fig. 8c). This result indicated that the functional polymer was well coated on the surface of Fe_3_O_4_@mSiO_2_.

As shown in Fig. 8d, the functional nanocomposites were modified on the surface of bacteria *via* amino groups, which were obviously indicated in TEM images (Fig. 8d), because the bacterial surface became rough and lean. As the inset of Fig. 8d shown, the magnetic separation of BFSM was examined to analyze the magnetism qualitatively. BFSM could be separated easily and completely about 2 minutes under the applied external magnetic field. In addition, BFSM could easily and steadily disperse in water by shaking. This result indicated that BFSM had superior strong magnetism and eminent dispersibility, which were helpful in practical applications.

### The degradation of hexavalent chromium

**Figure 9:**
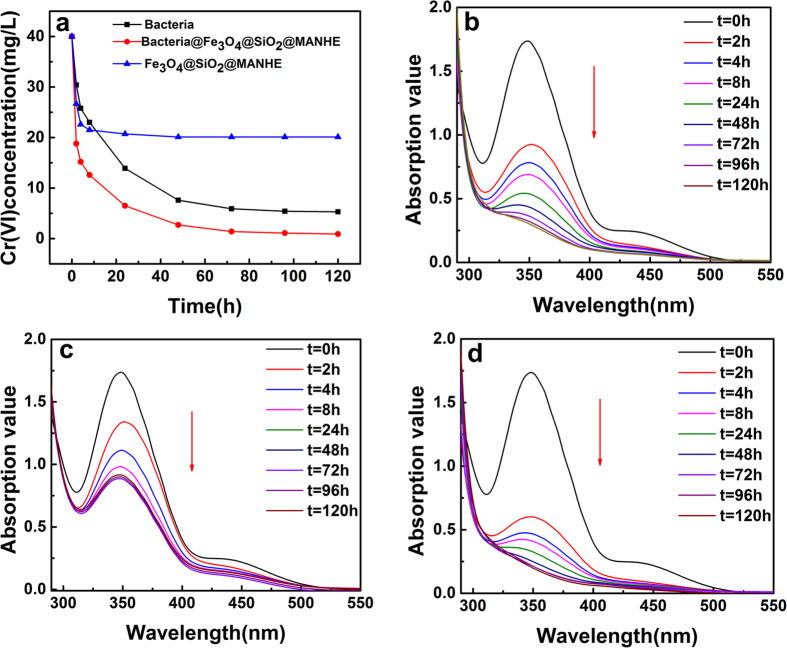
Contrast the reduction ability of 40 mg/L Cr(VI) (**a**); UV-vis of Cr(VI) by *B. subtilis* degradation (**b**); FSM (**c**); BFSM (**d**).

To study the ability of bionanocomposite to degrade hexavalent chromium, *B. subtilis*, FSM and BFSM were prepared to degrade 40 mg/L solution of hexavalent chromium with the same time under the optimal conditions respectively. The results were shown in Fig. 9a, the planktonic cells of *B. subtilis* degrade hexavalent chromium with the fastest rate in solution within 0 h to 8 h. Then, the concentration of hexavalent chromium would achieve equilibrium after 70 h. The removal efficiency of hexavalent chromium would reach 83.4% after 120 h. On the other hand, the concentration of hexavalent chromium would reach equilibrium after 4 h when treated by FSM. Meanwhile, only 50% of hexavalent chromium would be degraded from the 40 mg/L hexavalent chromium solution after 120 h. When treated by BFSM, it only spend 2 h for 50% removal of hexavalent chromium, and the equilibrium achieved after 96 h. it was noted that 94% of hexavalent chromium was removed from the 40 mg/L hexavaient chromium solution after 120 h. The UV-vis spectrum of 40 mg/L of hexavaient chromium treated by *B. subtilis*, FSM and BFSM were shown in Fig. 9 respectively (panels b, c and d). It is observed that the BFSM is more efficient in reduction of hexavaient chromium than the *B. subtilis* (Fig. 9b) and FSM (Fig. 9c). Because the UV-vis spectrum of hexavalent Cr-diphenylcarbazide complex was shown in Fig. 9d at 365 nm. It could be clearly seen that the adsorption value decrease more quickly than *B. subtilis* (Fig. 9b) and FSM (Fig. 9c). The Fig. 9b showed that there were no longer absorption band of hexavalent chromium centered at 365 nm for 120 h. The result was also in agreement with the Fig. 2b. So we can consider that the concentration of hexavalent chromium is close to zero according to results shown in Fig. 9b. As seen in Fig. 9c, the concentration of hexavalent chromium had no change after 24 h and achieved absorption equilibrium. However, no absorption band of hexavalent chromium centered at 365 nm was observed and the adsorption value even decrease to zero after 72 h. In this process of degradation, absorption and degradation of Cr(VI) would be combined into continuous process: (1) The BFSM surface of the absorptive polymer would efficiently bind Cr(VI), and then Cr(VI) absorbed on the surface of *B. subtilis*. (2) The degradation process would be promoted due to a higher local concentration of Cr(VI) on the surface of *B. subtilis*. Through the above comparison, the result strongly demonstrate the degradation of hexavalent chromium (Fig. 9), which further confirm the degrade ability of BFSM.

## Conclusions

In this study, an advanced bionanocomposites was prepared for removing the hexavalent chromium from wastewater. A core-shell structured, magnetic nanocomposite modified by functional polymer was prepared for local enriching the low concentrations hexavalent chromium. Then the core-shell structured, magnetic nanocomposite was connected on the surface of bacteria *via* amines on bacterial (*Bacillus subtilis* ATCC-6633) surface. After the immobilization, the adsorption-biodegradation process could be illustrated with two stages. In the first stage, the adsorption process played a main role and the hexavalent chromium concentration could increase relatively around the bacteria in a short time. And then, the biodegradation started in the second stage and completed the whole process. BSFM spent nearly 72 h achieving 94% the degradation efficiency of hexavalent chromium. In the meantime, the BFSM could be separated from the wastewater via its magnetism. Therefore, this composited technique can be potentially applied in the treatment of low-concentrated Cr(VI)-containing wastewater.

## Methods

### Materials

Ethylene glycol (EG), anhydrous sodium acetate (NaAc), ammonia solution (NH_3_·H_2_O, 28 wt.%), iron nitrate (Fe(NO_3_)_3_·9H_2_O), cetyltrimethylammonium bromide (CTAB), tetraethylorthosilicate (TEOS) were obtained from Sinopharm Chemical Reagent Co. Ltd. (Shanghai); Potassium dichromate (K_2_Cr_2_O_7_) and Dlphenylcarbazide were purchased from Aldrich; 4,4-Azobis(4-cyanovaleric acid) (ABCPA, 97%) and N-Acryloxysuccinimide was purchased from Sigma; Tryptone and yeast extract were supplied with Suzhou Biogene Biotechnology Co., Ltd. *B. subtilis* ATCC-6633 was obtained from Fujian Institute of Microbiology, China. All reagents were used as received without further purification.

### Synthesis of spherical Fe_3_O_4_

In this experiment, all the chemical agents were of analytical grade and were used without further purification. The spherical magnetic particles were prepared according to the literature with some modification^42^. As usually, 2.02 g of Fe(NO_3_)_3_·9H_2_O and 4.1 g of sodium acetate were dissolved in 50 mL of ethylene glycol (EG) with stirring for 30 min. The obtained solution was transferred to a Teflon-lined stainless-steel autoclave and heated at 180 °C for 6 h. Then the autoclave was naturally cooled to room temperature. The gained black magnetite particles were washed with ethanol for several times, and dried in vacuum at 60 °C for 5 h.

### Synthesis of Fe_3_O_4_@mSiO_2_

The core–shell structured Fe_3_O_4_@mSiO_2_ microspheres were prepared through a modified Stöber method. In a typical process, 0.10 g of obtained Fe_3_O_4_ particles were treated using 0.1 M HCl solution by ultrasonication for 20 min. Whereafter, the treated Fe_3_O_4_ particles were separated via centrifugation, washed with deionized water. At the same time, The Fe_3_O_4_ was dispersed in the mixture solution of 80 mL of ethanol, 20 mL of deionized water, and 1.0 mL of concentrated ammonia aqueous solution (28 wt.%). Afterward, 0.3 g of cetyltrimethylammonium bromide (CTAB) was added dropwise to the solution. After this, 0.25 mL TEOS was added dropwise into the solution under vigorous stirring for 6 h. After reaction for 6 h, the product was collected by magnetic separation and tautologically washed with ethanol and deionized water. The above coating process was redone twice. The structure-directing agent (CTAB) was removed with ethanol and deionized water for three times. The obtained precipitate was separated and washed with deionized water. Subsequently, the product was dried in vacuum at 60 °C for 24 h. The manufactured microspheres what was called Fe_3_O_4_@mSiO_2_.

### Synthesis of Fe_3_O_4_@mSiO_2_-NH_2_

200 mg (Fe_3_O_4_@mSiO_2_) of the nanoparticles obtained for 250 ml flask, the flask to add 150 ml acetonitrile, ultrasound 30 min, then add 3 ml KH550, mechanical agitation for the night.

### Synthesis of Fe_3_O_4_@mSiO_2_@MANHE

The acid chloride derivative of ABCPA (Cl-ABCPA) was prepared by a reaction of ABCPA and PCl_5_. ABCPA (3.0 g) was dissolved in dichloromethane (25 mL) and cooled to 0 °C. PCl_5_ (24 g) in 25 mL of CH_2_Cl_2_ was added into the above solution and stirred overnight. After the reaction, the excess PCl_5_ was removed by filtration. The clear solution was added into 5-fold of hexane at 0 °C, and 4,4-azo-bis(4-cyanopentanoicchloride) was obtained after filtration. Fe_3_O_4_mSiO_2_-NH_2_ nanoparticles (0.600 g) were added to 80 mL of dry dimethylformamide. After 0.5 h of ultrasonication, Fe_3_O_4_@mSiO_2_-NH_2_ (0.60 g) was dispersed in a mixture of 80 mL of CH_2_Cl_2_ and 2 mL of triethylamine, and Cl-ABCPA (2.5 g) in 25 mL of dry CH_2_Cl_2_ was added to the dispersion. After stirring at 0 °C for 2 h, the dispersion was stirred at room temperature overnight. Fe_3_O_4_@mSiO_2_-ABCPA was obtained after filtration and washing with methanol and dichloromethane. Polymer on Fe_3_O_4_@mSiO_2_-ABCPA sheets were prepared by free-radical polymerization. In a Schlenk flask, Fe_3_O_4_@mSiO_2_-ABCPA (0.05 g), 4-vinylpyridine (4 mL) and N-Acryloxysuccinimide (0.7 g) monomer were dissolved in 9 mL of Cyclohexanone. After 0.5 h min sonication, the dispersion was stirred at 75 °C for 5 h. The resulting product was dissolved in acetone and centrifuged to remove the free polymer chains which were not anchored to the nanoparticles. The final product (FSM) was dried in vacuum at 50 °C.

### Bacteria cultivation

*B. subtilis* ATCC-6633 was obtained from Fujian Institute of Microbiology, China. Previous study suggested that the maximum hexavalent chromium resistance of *B. subtilis* ATCC-6633 is 40 mg/L. Planktonic cells were grown at 30 °C with shaking (120 rpm) for 48 h in modified LuriaeBertani (LB) liquid medium (pH=7) supplemented with 5 g/L NaCl, 10 g/L tryptone, 5 g/L yeast extract and 5 g/L glucose.

### Synthesis of BFSM

After strain *B. subtilis* ATCC-6633 was cultured for 48 h in 100 mL LB medium with shaking (120 rpm), the *B. subtilis* ATCC-6633 were harvested by centrifugation (5 min, 5500 g) and washed twice with PBS (sterile phosphate buffer solution). Then, the cell pellets were resuspended in PBS. Subsequently, 30 mg FSM was added into above system. Planktonic cells were cultivated at 30 °C with shaking (120 rpm) for 5 h. After that, BFSM were obtained by magnetic separation.

### Chromium reduction and immobilization experiments

In order to prepare the chromium stock solution, K_2_Cr_2_O_7_ (AR) was dissolved in deionized-distilled water. In the experiments, the initial hexavalent chromium concentration was 40 mg/L. Cr-bacteria interaction experiments were conducted in two steps as follows: 1) planktonic cells were cultured in LB medium for 48 h and then harvested by centrifugation at 5500 g for 5 min; 2) bacteria were transferred into 100 mL glucose solution (5 g/L, pH = 7) with a final concentration of 0.01 g/mL, and bacteria-Cr interaction experiments were conducted by adding chromium stock solution to the designed initial concentration at pH 7.0. At the same time, BFSM- Cr interaction experiments were conducted by adding chromium stock solution to the designed initial concentration under optimal conditions. At last, samples were periodically taken with a sterile needle and a syringe for the analysis of the Cr species.

### Analytical methods

The UV-vis absorbance spectrum of hexavalent chromium and the concentrations of hexavalent chromium were obtained using a Shimadzu UV 3600 spectrophotometer equipped with an equipped with a MPC-3100 integrating sphere attachment. The total concentrations of Cr were obtained using atomic absorption spectrophotometer (PinAAcle 900T). The size of the bacteria and surface of bacteria were examined by scanning electron microscope (SEM; Hitachi S-4800). Transmission electron microscopy (TEM; Hitachi H600) was used to observe the transformation of nanoparticles in the synthesis process; Fourier transform infrared spectroscopy (FT-IR; Nicolet 4700) was employed to represent results after wrapping SiO_2_. The thermal properties of the composites were measured by thermogravimetric analysis (TGA). The samples were heated to 800 °C at a heating rate of 10 K/min under nitrogen atmosphere on a Netzsch TG209 instrument. X-ray diffraction (XRD) (X’Pert-Pro MPD) was taken to analyze the crystal phase.

## Additional Information

**How to cite this article**: Zhong, Y. *et al*. Fabrication of Unique Magnetic Bionanocomposite for Highly Efficient Removal of Hexavalent Chromium from Water. *Sci. Rep*. **6**, 31090; doi: 10.1038/srep31090 (2016).

## Figures and Tables

**Figure 1:**
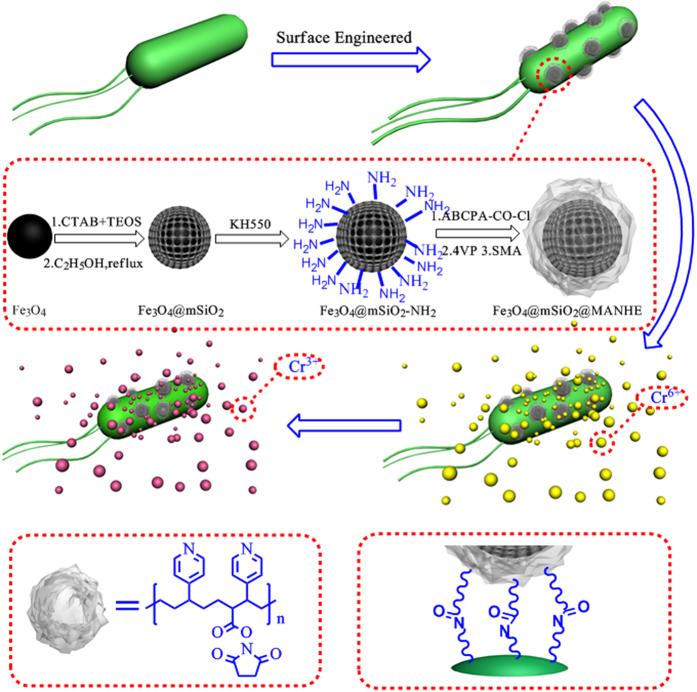
Schematic illustration of the synthetic procedure for BFSM and reduction of Cr(VI) into Cr(III).
